# Influence of Different Microplastic Forms on pH and Mobility of Cu^2+^ and Pb^2+^ in Soil

**DOI:** 10.3390/molecules27051744

**Published:** 2022-03-07

**Authors:** Agnieszka Medyńska-Juraszek, Bhakti Jadhav

**Affiliations:** Institute of Soil Science, Plant Nutrition and Environmental Protection, Wroclaw University of Environmental and Life Sciences, 53 Grunwaldzka Str., 50-357 Wrocław, Poland; bhakti.jadhav@upwr.edu.pl

**Keywords:** microplastic, fibers, particles, soil properties, pH, heavy metals

## Abstract

Microplastics, due to their surface properties, porosity and electrostatic interactions have a high affinity for cations sorption from the aqueous phase. As soil is a complex matrix, interactions between microplastics, soil constituents and heavy metals (HM) may modify the soil microenvironment for heavy metal mobilization/immobilization processes. In order to better understand the problem, three commonly found forms of microplastics in soil (fibers, fragments and microbeads) were mixed with Cu^2+^- or Pb^2+^-contaminated soil and incubated at 22 °C for 180 days. In soil samples pH and the content of water and acid exchangeable species of metals were analyzed. The results of this study showed that the presence of microplastics in HM-contaminated soil affected metal speciation, increasing the amount of easily exchangeable and potentially bioavailable forms of Cu^2+^ or Pb^2+^ in the tested soil. Soil pH also increased, confirming that microplastic particles affect soil properties relevant to the sorption/desorption process of metal cations. Overall, the smallest microplastic particles (≤1 mm), such as fibers or glitter microbeads, had a greater impact on the change in the sorption and desorption conditions of metals in tested soil than larger particles. The findings of our study show that microplastic form, shape and size should be considered as important factors that influence the soil properties and mobility of heavy metals in soil.

## 1. Introduction

Microplastics (MPs), defined as particles with a size of less than 5 mm, became a threat of global concern due to their unknown, potentially negative impacts on the environment. Due to their small particle size and very slow biodegradation rate, they can be easily absorbed by organisms and, subsequently, transported through food webs [1,2]. Compared with large forms of plastic, microplastics exhibit a large specific surface area, high hydrophobicity [3,4] and remarkable binding capacity [5] and may act as a vector for other organic and inorganic contaminants in environmental media [6]. The majority of previous studies focused on contaminants and microplastic interactions in waters, e.g., storm waters [7], marine waters [3] or water sediments [8], showing that MPs can play a key role as a vector of chemical contaminants in marine ecosystems [9]. MPs are confirmed to have a high affinity to heavy metals from the aqueous phase [10], rapidly adsorbing heavy metals from nearby metal sources [11]. Much less is known about MPs in terrestrial ecosystems; nonetheless, microplastic pollution in soil is considered as an important point source of contaminants in aquatic ecosystems [12]. Agricultural soils potentially store more microplastics than oceanic basins [13]. Recent estimates reported that the annual input of microplastics by farmlands ranges from 63 to 430 thousand tons in Europe and 44 to 300 thousand tons in North America, both of which exceed the extrapolated annual emissions of microplastics into ocean surface waters [14]. Microplastics in soil occur in two types: primary and secondary. Primary microplastics are mainly represented by microbeads, plastic pellets or fibers manufactured in sizes smaller than 5 mm and directly incorporated into the environment as an airborne or wastewater deposition [15]. Secondary microplastics form from the breakdown of larger plastics due to weathering or biodegradation processes [16]. It is reported that most microplastic in soil originate from polyethylene and polypropylene plastics [17]. Synthetic fibers (including nylon and polyester) produced by wastewater treatment and the application of sewage sludge are the predominant microplastic type occurring in soil (up to 92%) [18]. Much less can be found in form of fragments (4.1%) occurring in soil due to the defragmentation of plastic waste or microbeads and nanoparticles (1.3%) [19]. Such diversity of MPs and the complexity of possible interactions between soil constituents, MPs and heavy metals have resulted in an increasing interest in the problem of MPs accumulation in soil. The adsorption of heavy metals on MPs may alter their environmental behavior, fate, bioavailability and toxicity. The indirect accumulation of MPs in soils may alter soil properties, e.g., soil porosity, water holding capacity [4], organic carbon content or microbial activity [20] modifying the soil microenvironment for heavy metal mobilization/immobilization processes. Some studies directly showed that MPs can adsorb heavy metals, and this could be an important factor governing the transformation of heavy metal speciation in soil [21]. Hodson et al. [22] found that Zn^2+^ adsorbed by plastic-derived microplastics could become more bioavailable to soil mesofauna, suggesting their environmental risk to terrestrial ecosystems. Little is known about the effects that microplastics of different shapes and forms may have on soil properties and the mobility and bioavailability of heavy metals. Different methods of microplastic pollution detection in soil have been implemented; however, optical microscopy and the manual recognition of MPs by forms, shapes and colors are the most common and easily available methods. Combining the data from a microscopy analysis with the contents of metal species in soil can be a useful and simple tool for predicting the potential risk related to the occurrence of MPs and chemicals in soil environments.

The aim of this study was to investigate the potential effects on Cu^2+^ or Pb^2+^ mobility and bioavailability in the presence of three commonly found in soil microplasticcontaminations: fibers, fragments and microbeads, representing primary and secondary MPs. To meet our objectives, soil was co-contaminated with metals and microplastics and incubated for 180 days. After soil incubation, samples were collected, and soil pH and the content of water-extractable and exchangeable forms of heavy metals were analyzed to evaluate the changes in Cu^2+^ or Pb^2+^ speciation.

## 2. Materials and Methods

### 2.1. Microplastic Characterization

Four microplastic forms of varying sizes and shapes were prepared for tests (Table 1). Microfibers were obtained during a drying process of polyester fleece blankets and sweatshirts in a tumble dryer, according to the procedure proposed by Selonen et al. [23]. Defragmented plastic bag particles were collected from decomposing material prepared by modified Compostable Product Test ASTM D6400 and EN13432. Briefly plastic bags were inoculated with compost leachates and incubated for 6 months in glass jars maintaining moisture, sun light and aerobic conditions. Plastic bottle was cut with scissors into pieces with diameters ranging from 1.0 to 5.0 mm, according to the procedure described by Lehmann et al. [24]. Glitter, representing the form of MPs present in a wide range of cosmetics and personal care products, was bought in an art shop. 

### 2.2. Soil Incubation Experiment

To avoid preferential sorption of Cu^2+^ and Pb^2+^ sandy soil with very low organic matter (0.43%) and clay minerals (2%) content was chosen for the incubation experiments (Table 2). To avoid microplastic contamination, soil was collected from a buried soil profile at the mining site and spiked with Cu^2+^ or Pb^2+^ metal ions and fibers, plastic bag or bottle particles, and glitter. The former sand and gravel mining site was located in the west part of Poland (N 51°49′19.46″, E 15°13′47.8″, 72 m a.s.l.) near Nowogród Bobrzański. Briefly, 50 g of soil was placed in a glass bottle and spiked with 5 mL of the standard solution, containing either 25 ppm of Cu^2+^ or Pb^2+^ in forms of CuNO_3_ or PbNO_3_ (TraceCERT^®^, in 10% nitric acid) purchased from Merck (Darmstadt, Germany), according to the sample spiking procedure described by Schwertfeger and Hendershot [25]. Spiked soils were incubated for 5 days under constant humidity to avoid the quick sorption of metals and for equilibration. Then, 25 mg of each microplastic was mixed with metal-spiked soils. The incubations were maintained at a consistent temperature (22 °C) and humidity (80%) for 180 days. The soil moisture level was maintained at 70% of the maximal water holding capacity of the soil during the incubation period.

### 2.3. Metal Analysis in Soil

Soil for analysis was collected once after incubation period. Total content of Cu and Pb in soil was analyzed after sample digestion in 65% nitric acid (1:10 *w/v* ratio) in microwave system StartD (Milestone, Sorisole, Italy), according to the EPA 3051A method. The content of water-extractable forms of Cu^2+^ and Pb^2+^ was obtained by soil sample shaking (1:4 ratio) with distilled mili-Q water for 24 h on orbital shaker according to modified method DIN 38414-S4 described by Shuwirth and Hofmann [26]. To determine exchangeable and easily soluble forms of Cu^2+^ and Pb^2+^, the first step of Community Bureau of Reference (BCR) sequential extraction procedure was performed. Briefly, a 1 g soil sample was extracted with 40 mL of 0.11 mol L^−1^ acetic acid by shaking the solution for 16 h [27]. Metal concentrations in digested and extracts were analyzed on Microwave Plasma-Atomic Emission Spectrometer MP-AES 4200 (Agilent Technologies, Santa Clara, CA, USA) after sample filtration on Munktell No. 2 filters, grade 0.84 g/cm^2^ (Ahlstrom Munksjö, Helsinki, Finland). To avoid analytical errors, standard solutions (from LGC Standards Ltd., Teddington, UK) for MP-AES 4200 and certified reference material RTH 953 Heavy Clay Soil from LGC Promochem (LGC Standards Ltd., Teddington, UK) were used for calibration, and the total Cu and Pb contents—41.1 and 11.9 mg/kg, respectively—were analyzed with every sample set. The recovery of Cu and Pb from certified reference material (CRM) was 87–92%, and the maximum values of RSD were 3.4%. Detection limits were 0.01 mg/kg of Cu and 0.02 mg/kg of Pb in the soil samples.

### 2.4. Statistical Analysis

All sorption experiments and measurements were performed in triplicates. The data are presented as the mean values with relative standard deviation (RSD). Student’s *t*-tests were used to test for significant differences in pH and metal–water exchangeable content between microplastic-spiked and control soil (*p* < 0.05). The obtained data were compiled using Microsoft Excel 2016. 

## 3. Results

### 3.1. Effect on Soil pH

The addition of MPs increased soil pH (Table 3). In Cu-spiked soil, pH increased after MPs application by 14.25% in FBS, 8.9% in PBS, 5.79% in PETS and 7.34% in GLS. Similarly, pH increased in Pb-spiked soil by 13.36% in FBS, 9.35% in PBS, 6.68% in PETS and 2.85% in GLS. In all treatments, expect for the treatment with PETS and Cu-spiked soil, soil pH changes were statistically significant.

### 3.2. Effects on the Metal Species in Soil

The highest contents of water-extractable Cu were determined in treatments with glitter and fiber—1.74 mg/kg and 1.70 mg/kg, respectively—while for Pb, the highest contents were determined in treatments with glitter and defragmented plastic bags (Table 4). Similar amounts of Cu^2+^ and Pb^2+^ were extracted when comparing each treatment, except FBS and Cu^2+^ and PBS and Pb^2+^, suggesting that the presence of plastic fibers (primary microplastics) can increase the leachability of Cu, while fragments of plastic bottles representing secondary microplastic can increase the leachability of Pb and impact the mobility of pollutants and its potential transfer from the solid to liquid phases (soil solution).

Compared with the amounts extracted with deionized water, 0.11 mol L^−1^ CH_3_COOH extracted up to three times more of Cu^2+^ and up to five times more of Pb^2+^. The presence of microplastics caused a significant increase in Cu and Pb in exchangeable forms compared with unpolluted soil (Table 5). However, the amount of extracted ex-metals depended on microplastic form and particle size. For both of the tested metals, the highest contents of easily exchangeable forms were determined in soil containing glitter (GS) with a particle size < 1 mm. The lowest content of ex-Cu and Pb was determined in soil containing larger particles of PET bottles. When comparing the difference between metals, similar amounts of both cations were extracted from soils containing fibers (FBS), while a significant increase in exchangeable forms of Pb^2+^ compared with Cu^2+^ was determined in soil containing microplastics from plastic biodegradable bags. 

**Figure 1:**
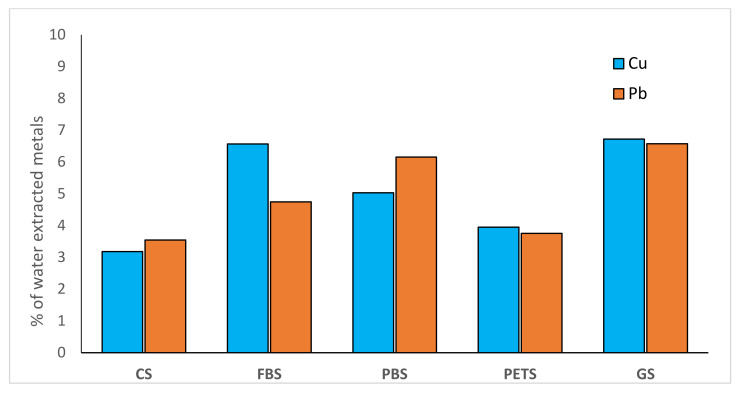
Water-extracted forms of heavy metals in microplastic polluted soils.

The results of the experiment showed that presence of MPs increased the participation of water-extractable Cu^2+^ and Pb^2+^ in soil up to 5.5% (mean value for all treatments) compared with 3.3% in microplastic-free soil (Figure 1). The highest increase in Cu^2+^ in water extract was observed in soil-containing fibers, and in Pb^2+^ in soil with microplastics derived from plastic bags (PBS). Water-extractable cations are considered to be mobile, representing the amount of metals that can be easily transferred from the solid to liquid phase; therefore, the obtained results suggest that the presence of microplastic can increase Cu and Pb leachability in soil.

**Figure 2:**
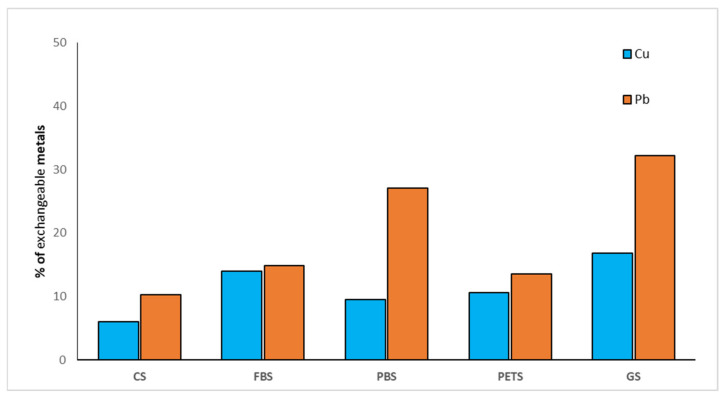
Exchangeable and easily soluble forms of heavy metals in microplastic-polluted soils.

In the presence of microplastic particles, the participation of exchangeable forms in the total content of metals increased. However, the changes were more significant for Pb^2+^ cations compared to Cu^2+^ (Figure 2). Respectively, 27% and 32% of Pb was in exchangeable form in PBS and GS treatments, compared to 10% in control soil without microplastics and 9% and 16% in the same treatments with Cu^2+^ spiking. Similar increases in both tested metals were observed for the FBS treatment—13% and 14%, respectively—while slightly higher amounts of Pb^2+^ compared to Cu^2+^ were determined in PBS.

## 4. Discussion

Recently, MPs were considered to be carriers for heavy metals in environmental media [28]. It was reported that metal cations, e.g., Cu^2+^ or Pb^2+^, exhibit various affinity towards MPs particles [29]. The mobility of metal cations is greatly contingent on plastics with varied physicochemical properties, e.g., polymer type [30], crystallinity, density or particle size [31], and specific surface area [3]. The adverse effects on biota and MPs’ potential activity as a pollutant carrier in the environment may depend on their sizes and shapes. For example, synthetic fibers induce stronger adverse effects on aquatic organisms than microbeads; while for smaller particles, the probability of ingestion increases [23,32]. The knowledge of possible interactions between microplastics and soil is still very limited. Since the number of fibers, microbeads and irregularly shaped microplastics introduced in soil is rapidly increasing, the need to develop state-of-the-art methods is urgent. Our results indicated that microplastics increased soil pH, causing a change in the soil microenvironment that had an important influence on the heavy metals sorption/desorption process. A higher soil pH decreases mobility and increases Cu^2+^ and Pb^2+^ adsorption; however, in our study, the increase in soil pH led to an increased adsorption of heavy metal ions by MPs. Similar findings were observed by Holmes et al. [33], describing that the sorption capacity of Pb^2+^ on high-density polyethylene increased with increasing pH; however, no clear trend for Cu^2+^ was observed in this study. This can be explained by the higher negative charge of the MPs surface in higher pH values, generating an electrostatic attraction to the metal cation [34]. Electrostatic attraction seems to play the most important role in divalent cation sorption. Zou et al. [35], studying the adsorption of Pb^2+^ and Cu^2+^ in four polyethylene forms (CPE, PVC, HPE and LPE), showed that Pb^2+^ electrostatic interaction played an important role in sorption, while electrostatic interaction and complexation determined the sorption of Cu^2+^. The findings of our study are also in agreement with Wu et al. [36], who showed that nanoparticles (PSNPs) mobility increased with the increase in soil pH polystyrene, and plastic particles could be transported in soil across further distances due to surface charges in the soil and PSNPs. Our results showed that the highest increase in pH was observed in soil with polyester fibers, which agrees with previous research of other authors [37]. De Souza Machado et al. [4] showed that the intensity and directions of the changes in soil properties depend on the microplastic type, and the increase in soil pH in the presence of MPs can be explained by the increase in soil aeration, porosity and the alternation of soil biota due to toxic compounds leaching from the plastic. The linear shape, size and flexibility of microfibers reduces soil aggregation and soil bulk density with a possible impact on soil structure and water retention [38]. Similar observation in soil polluted with different MPs types was also described by Zhao et al. [37], showing that, depending on the shape, polymer type and exposure time, microplastics can have different effects on soil properties; however, the increase in pH is mostly determined after longer MPs exposure in soil.

The results of the experiment showed that, in the presences of microplastic particles in soil, Cu^2+^ and Pb^2+^ leachability increased, causing a risk of contaminant migration in soil. The explanation of this finding can be related to pH changes in soil and electrostatic interactions. Hydrogen bonds are specific weak electrostatic interactions, involving the hydrogen ion H^+^ and can affect the sorption on polymers when proton donor and proton acceptor groups are involved [39]. Other interactions promoting the sorption of chemicals onto MPs are van der Waals forces and, as very weak interactions occurring between the molecules, they can be easily broken by water. If the indicated mechanisms are dominant in the HM sorption process in MPs, the strength of thses bonds might be weaker and less stable compared to bonds formed between soil organic matter (SOM) and HM; therefore, metal cations were more easily desorbed from MPs’ surfaces, contributing to the process of a more intensive leachability of Cu and Pb in tested soils. Medium proprieties may also influence sorption by modifying microplastics surface charge. In soil, MP surfaces can become negatively charged due to pH changes or by interactions with dissolved organic matter (DOM), e.g., humic acids forming “super molecules” with a higher surface area, more anionic sites for metal sorption and electrostatic synergy for cations binding [40,41]. Free cations are likely to react with charged regions of negative MP surfaces, which can be created by the adsorption of organic molecules [10,39,42]. MPs facilitates the solubility and mobility of metals, and thus increase the transport of pollutants [20], similar to DOM, as microplastic molecules have very similar properties to natural organic matter, including a large surface area, sorption capacity for cations and diverse functional groups (e.g., C-H, C-N, C=O and N-H) [5]. This suggests that competition between soil particles, e.g., organic matter or clay minerals and microplastics, is more likely to occur in soil than in other environmental media. MPs’ interactions with dissolved organic matter (DOM) play a crucial role in MPs transport in soil and possible implications to its toxicity. MP pollutants impact microbial soil decomposition, reducing soil-dissolved organic carbon (DOC) content; however, it remains unclear whether MPs inhibit the release of DOC from SOM or accelerate the decomposition of pre-existing DOC [43,44]. The distinction between the sorption process occurring in MPs and SOM can be difficult, as the multi-step procedure of microplastic extraction can remove metal cations from the polymer surface. Scanning electron microscopy (SEM) with XRD might be helpful in this process; however, high cost and inaccessible technology limits the use of this method, and there is an urgent need to develop new methods of problem analysis. Performing the first step of the BCR sequential extraction showed that microplastics, when present in soil, are able to shift metal cations from residual to easily exchangeable/mobile forms, causing a risk of pollutant mobilization and transfer to the food chain by plant and soil biota uptake. Similar findings were described by Yu et al. [21], studying the chemical speciation of Cu, Cr and Ni in soil spiked with polyethylene particles, suggesting that, in coarse and large particle materials, the presence of MPs causes more visible changes in metal speciation and the partial sorption of metal cations in MP particles. The results of the experiment showed that, in presence of microplastics, the amount of metals in exchangeable and potential bioavailable forms increased significantly. However, more relevant changes were observed for Pb^2+^, especially in the presence of microplastics derived from biodegradable plastic bags and tiny glitter particles (from PE polymers). This observation is in agreement with studies of Han et al. [45], describing that PET microplastics have a relatively rapid and strong ability to adsorb Cu(II), and PE microplastic has the relatively rapid and strong ability to adsorb Pb(II). Nonetheless, our results show that polyester fibers play a more significant role in the sorption/desorption process of Cu^2+^ compared to other tested forms of MPs. 

Indicated differences in metal mobility in tested sandy soil confirmed that adsorption behavior varies between types of MPs exhibiting distinct surface physicochemical properties. Godoy et al. [29] described that specific surface, porosity and morphology are characteristics of the plastics that influence adsorption. In our study, microplastics obtained from PET (polyethylene phthalate) bottles had the least impact, while polyethylene (PE) and polyester (PEs) in fibers contributed more to the Cu^2+^ and Pb^2+^ sorption process. This can be explained by different properties of polymers. PET has a lower specific surface area and smaller adsorption capacity than polystyrene or PVC [46]. Polyesters, polyamide and polyacrylonitrile fibers are used to produce adsorptive materials for heavy metal removal from water [47,48] and we assumed that PEs fibers may be characterized with higher specific surface area and adsorption capacity. Asaad et al. [49] described that polyesters exhibit high biding capacities for Cd^2+^, Cu^2+^ and Pb^2+^; however, there is a gap in knowledge of potential impacts of fibers on heavy metals mobility in soil. The findings of our study are also in agreement with Han et al. [45], who confirmed that the adsorption capacity of Cu^2+^ and Pb^2+^ can be affected by microplastic types, but also that in metal species, including both tested metals, Pb^2+^ can be adsorbed in larger amounts. Finally, the size of the particle is also a very important characteristic of the MPs, determining their possible mobility and reactivity in environmental media. The effect of particle size on the HM adsorption process is still under discussion. Small-particle-size MPs can provide a larger specific surface area and more relevant adsorption sites, which are active in the process surface functional groups [5,11,35]. In contradiction, Tourinho et el. [39] stated that the size and shape are less important in the HM adsorption process, as absorption does not depend on the availability of sorption sites on the surface but mainly on the area-to-volume ratio of an MP particle. Liu at al. [50] described that particles presenting a higher area-to-volume ratio have a higher adsorption capacity, e.g., small size particles (<1 mm) or particles with irregular shapes [41]. Although the surface properties of MPs, e.g., specific surface area, or surface functional groups were not determined in our study, the hypothesis that area-to-volume ratio is relevant for describing MP sorption capacity seems to be indicated in the described experiment. Our results proved this concept, showing that small-size particles such as glitter microbeads or MPs with irregular shapes, e.g., decomposed plastic bag particles and fibers, will be more active in the mobilization process of HM in soil compared to larger plastic particles, e.g., defragmented plastic bottle. Nevertheless, the plastic ageing process occurring in soil due to weathering, oxidation and defragmentation will cause uneven surface–structure changes, in most cases increasing MPs’ sorption capacity [51]. This should be considered when predicting the potential impacts of MPs’ presence in heavy metal co-contaminated soils. As fibers and plastic bag debris are the types of MPs mostly occurring in soil, more attention should be paid to possible changes in soil due to the presence of this specific contamination.

## 5. Conclusions

The study contributes to a better understanding of the effects that microplastics particles have on heavy metal mobility and bioavailability in soil. Our results showed that the long-term deposition of microplastic in soil increases soil pH, modifying conditions of heavy metal mobilization/immobilization processes. The increase in soil pH usually causes metal immobilization, decreasing the risk of pollutant transfer in the food chain. However, our results indicated the opposite effect, and in the presence of microplastic particles, the Cu^2+^ and Pb^2+^ leachability/mobility increased significantly. Moreover, this study indicated a higher content of acid-exchangeable forms of tested metals in MPs-polluted soil, suggesting that MPs particles contributed to the process of Cu^2+^ and Pb^2+^ adsorption, which affected metal speciation in the soil. The effect on metal speciation and mobility in soil can be related to MPs physicochemical properties; however, metal specification should also be considered. Pb^2+^ cations were most likely affected by the presence of microplastics in soil compared to Cu^2+^, and almost double the contents of Pb^2+^ was indicated in soils containing fibers and glitter microbeads. Considering the polymer type, results of our study suggest that polyester- (PEs) and polyethylene (PE)-derived microplastics were most likely to mobilize Pb^2+^ compared to polyethylene-terephthalate (PET)-derived particles. However, more relevant changes in metal leachability and exchangeable forms were indicated with different particle shapes and sizes, showing that irregular-shaped particles, such as MPs derived from biodegradable plastic bags or small-size particles (<1 mm) characterized by a higher area-to-volume ratio, contributed more to the process of metal mobilization. The findings of our study showed that microplastics particles could potentially have a significant effect on the fate of heavy metals in soil; however, further investigations our necessary to understand this problem.

## Figures and Tables

**Table 1:** Description of the microplastics particles used in the experiment.

Microplastics Form	Polymer	Shape	Size Range
Fibers (FB)	Polyester (PEs)	Lines	0.3 mm–3.0 mm
Plastic bag (PB)	Biodegradable polyethylene	Fragments with sharped edges	0.5–4.5 mm
Plastic bottle (PET)	Polyethylene terephthalate PET	Squares	1.0–5.0 mm
Glitter (G)	Polyethylene	Microbeads	0.5–1.0 mm

**Table 2:** Characteristics of soil used for the experiment.

Characteristics	Value
Texture	loose sand (92% sand, 5% silt, 2% clay)
pH in distilled water	4.49
EC *	1.23 µS cm^−1^
CEC	1.04 cmol(+) kg^−1^
TOC	0.43%
TN	0.12%
Total Cu	0.82 mg kg^−1^
Total Pb	0.43 mg kg^−1^
Cu_H20ex_ Pb_H2Oex_	0.05 mg kg^−1^ <0.02 mg kg^−1^

* EC—electric conductivity, CEC—cation exchange capacity, TOC—total organic carbon. TN—total nitrogen, Total Cu and Pb—after sample digestion in 65% nitric acid, H_2_Oex—water-extractable

**Table 3:** Soil pH after incubation with microplastics.

Treatment	Cu-Spiked Soils	Pb-Spiked Soils
CS *	4.49 ** ± 0.07 ***_a_	4.49 ± 0.14 _a_
FBS	5.13 ± 0.06 _b_	5.09 ± 0.01 _b_
PBS	4.89 ± 0.12 _b_	4.91 ± 0.17 _b_
PETS	4.75 ± 0.02 _a_	4.79 ± 0.14 _b_
GLS	4.82 ± 0.16 _b_	4.62 ± 0.17 _b_

* CS—control soil without microplastics, FBS—soil with microfibers, PBS—soil with microplastics derived from plastic bag, PETS—soil with microplastics derived from plastic bottle, GLS—soil with microplastic glitter particles; ** mean value (*n* = 3); *** standard deviation (SD). Different lowercase letters (_a_ and _b_) indicate significant differences between microplastic-spiked and control soil within each microplastic type (*p* < 0.05).

**Table 4:** Water-extracted forms of metals in microplastic-polluted soil.

Treatment	Cu mg kg^−1^	Pb mg kg^−1^
CS *	0.82 ** ± 0.10 *** _a_	0.9 ± 0.06 _a_
FBS	1.70 ± 0.06 _b_	1.21 ± 0.08 _b_
PBS	1.30 ± 0.18 _b_	1.57 ± 0.12 _b_
PETS	1.02 ± 0.22 _b_	0.96 ± 0.12 _b_
GS	1.74 ± 0.26 _b_	1.68 ± 0.24 _b_

* CS—control soil without microplastics, FBS—soil with microfibers, PBS—soil with microplastics derived from plastic bag, PETS—soil with microplastics derived from plastic bottle, GS—soil with microplastic glitter particles; ** mean value (*n* = 3); *** standard deviation (SD). Different lowercase letters (_a_ and _b_) indicate significant differences between microplastic-spiked and control soil within each microplastic type (*p* < 0.05).

**Table 5:** Exchangeable forms of metals in microplastic-polluted soils.

Treatment	Cu mg kg^−1^	Pb mg kg^−1^
CS *	1.57 ** ± 0.11 *** _a_	2.62 ± 0.08 _a_
FBS	3.63 ± 0.09 _b_	3.78 ± 0.08 _b_
PBS	2.46 ± 0.08 _b_	6.92 ± 0.08 _b_
PETS	2.74 ± 0.12 _b_	3.45 ± 0.09 _b_
GS	4.36 ± 0.06 _b_	8.23 ± 0.14 _b_

* CS—control soil without microplastics, FBS—soil with microfibers, PBS—soil with microplastics derived from plastic bag, PETS—soil with microplastics derived from plastic bottle, GS—soil with microplastic glitter particles; ** mean value (*n* = 3); *** standard deviation (SD). Different lowercase letters (_a_ and _b)_ indicate significant differences between microplastic-spiked and control soil within each microplastic type (*p* < 0.05).

## Data Availability

Data sharing is not applicable to this article.

## Sample Availability

Samples of the microplastics and soil samples are available from the authors.
